# Proteins of TNF-α and IL6 Pathways Are Elevated in Serum of Type-1 Diabetes Patients with Microalbuminuria

**DOI:** 10.3389/fimmu.2018.00154

**Published:** 2018-01-31

**Authors:** Sharad Purohit, Ashok Sharma, Wenbo Zhi, Shan Bai, Diane Hopkins, Leigh Steed, Bruce Bode, Stephen W. Anderson, John Chip Reed, R. Dennis Steed, Jin-Xiong She

**Affiliations:** ^1^Center for Biotechnology and Genomic Medicine, Augusta University, Augusta, GA, United States; ^2^Department of Obstetrics and Gynecology, Medical College of Georgia, Augusta University, Augusta, GA, United States; ^3^Department of Medical Laboratory, Imaging, and Radiologic Sciences, College of Allied Health Sciences, Augusta University, Augusta, GA, United States; ^4^Atlanta Diabetes Associates, Atlanta, GA, United States; ^5^Pediatric Endocrine Associates, Atlanta, GA, United States; ^6^Southeastern Endocrine & Diabetes, Atlanta, GA, United States

**Keywords:** inflammation, cytokines, cytokine receptors, diabetes, microalbuminuria

## Abstract

Soluble cytokine receptors may play an important role in development of microalbuminuria (MA) in type-1 diabetes (T1D). In this study, we measured 12 soluble receptors and ligands from TNF-α/IL6/IL2 pathways in T1D patients with MA (*n* = 89) and T1D patients without MA (*n* = 483) participating in the PAGODA study. Twelve proteins in the sera from T1D patients with and without MA were measured using multiplex Luminex assays. Ten serum proteins (sTNFR1, sTNFR2, sIL2Rα, MMP2, sgp130, sVCAM1, sIL6R, SAA, CRP, and sICAM1) were significantly elevated in T1D patients with MA. After adjusting for age, duration of diabetes, and sex in logistic regression, association remained significant for seven proteins. MA is associated with increasing concentrations of all 10 proteins, with the strongest associations observed for sTNFR1 (OR = 108.3, *P* < 10^−32^) and sTNFR2 (OR = 65.5, *P* < 10^−37^), followed by sIL2Rα (OR = 12.9, *P* < 10^−13^), MMP2 (OR = 5.5, *P* < 10^−6^), sgp130 (OR = 5.2, *P* < 10^−3^), sIL6R (OR = 4.6, *P* < 10^−4^), and sVCAM1 (OR = 3.3, *P* < 10^−4^). We developed a risk score system based on the combined odds ratios associated with each quintile for each protein. The risk scores cluster MA patients into three subsets, each associated with distinct risk for MA attributable to proteins in the TNF-α/IL6 pathway (mean OR = 1, 13.5, and 126.3 for the three subsets, respectively). Our results suggest that the TNF-α/IL6 pathway is overactive in approximately 40% of the MA patients and moderately elevated in the middle 40% of the MA patients. Our results suggest the existence of distinct subsets of MA patients identifiable by their serum protein profiles.

## Introduction

Chronic hyperglycemia is considered to be the main pathogenic factor involved in microalbuminuria (MA). Inflammation, increased apoptosis, and abnormal activation of the endothelium are increasingly thought to be important mechanisms for the development of MA (1). Pro-inflammatory pathways related to TNF-α and IL6 signaling can lead to enhanced deposition of extra-cellular matrix (ECM) within kidneys as well as activation of T cells, leukocytes, and endothelial cells in diabetic patients with MA (2, 3). In addition, inflammatory protein levels in the serum have been associated with the presence of MA in type-1 diabetes (T1D) patients (4–7).

The majority of the prior studies on human patients have been limited to measurement of a single or a few serum proteins in T1D patients with MA. Inflammatory proteins such as CRP, IL6, TNF-α, and the soluble form of TNF receptors (sTNFR1 and sTNFR2) as well as adhesion molecules have been individually examined in separate studies (4–8). In the EURODIAB study, elevated plasma concentrations of inflammatory markers were highly correlated with increased levels of clinical markers of microvascular complications (7, 9). In the prospective CARE study, elevated levels of sTNFR2 and CRP were associated with early renal function loss in subjects with chronic kidney disease (CKD) (5). A report from the Multi-Ethnic Study of Atherosclerosis showed significant associations between CKD and several proteins including CRP, IL6, sTNFR1, sICAM1, fibrinogen, and factor VII (10, 11). High serum concentrations of TNF-α, IL6, CRP, osteoprotegerin, fibrinogen, sICAM1, sTNFR2, and myeloperoxidase were shown to be associated with CKD status, higher cystatin C quartiles, and higher urinary albumin-to-creatinine ratio quartiles (7, 12). Recently, serum levels of sTNFR1 and sTNFR2 in fourth quartile were found to be predictive of early renal function loss in T1D patients (13). Along with proteins of the TNF-α pathway, proteins involved in endothelial cell activation, T-cell activation, and cellular differentiation were also shown to be involved in the progression of nephropathy (14). Serum levels of these proteins have been extensively studied and their association has been established in T1D patients with MA; however, these proteins have been rarely studied in the same cohort. Secondly, the single-protein analysis has been contradictory in previous studies. Therefore, it is unknown whether and how these proteins are co-regulated and whether certain combinations of proteins can serve as better biomarkers for MA.

In the current study, we investigated 12 serum proteins related to inflammation (CRP and SAA), endothelium activation and adhesion (sICAM1 and sVCAM1), immune and cellular activation (sTNFR1, sTNFR2, sIL2Rα, sgp130, and sIL6R), and ECM modeling enzymes (MMP1, MMP2, and MMP9) in T1D patients with and without MA. We demonstrate that multiple serum proteins in the TNF-α/IL6 pathway are significantly elevated in MA patients compared with T1D patients without MA. The prime goal of the study was to identify combinations of serum proteins that are even more strongly associated with the presence of MA in the T1D patient population and can be used to define subsets of MA patients with distinct inflammation patterns.

## Materials and Methods

### Study Population

All serum samples analyzed in the study were obtained from Caucasian subjects recruited into the Phenome and Genome of Diabetes Autoimmunity (PAGODA) study between 2002 and 2010. These subjects attended the Augusta University (AU) Medical Center and other endocrinology clinics in Augusta and Atlanta area of Georgia. T1D patients were screened for presence of microalbumin and creatinine in a random spot urine collection at the time of visit. Presence of MA was determined by the attending physician/endocrinologist, based on the last three microalbumin/creatinine ratio (MACR) values. We used the MACR <30 for T1D patients, and MACR values 30–300 for T1D patients with MA. Medical history, clinical, and demographic profiles for T1D subjects were captured from the medical charts (Table S1 in Supplementary Material). The research was carried out according to The Code of Ethics of the World Medical Association (Declaration of Helsinki). All study participants gave written informed consent. The study was reviewed and approved by the institutional review board at AU.

Blood samples were collected in clot activator tubes, allowed to clot at room temperature for 30 min prior to centrifugation at 3,000× *g*. Separated serum was then aliquoted into wells of 96-well plate to create a master plate. Individual daughter plates were then created by aliquoting 5–10 µL of serum from this master plate. All master and daughter plates were stored at −80°C until use.

### Laboratory Measurements

We selected to measure 12 proteins (CRP, SAA, MMP1, MMP2, MMP9, sgp130, sICAM1, sVCAM1, sIL2Rα, sIL6R, sTNFR1, and sTNFR2) in our PAGODA study subjects; the selection was based on published literature on role of these proteins in inflammation and MA. Luminex assays for these 12 proteins were obtained from Millipore Inc., Billerica, MA, USA. Multiplex assays were performed according to the instructions provided with the kit. Briefly, serum samples were incubated with antibody coated microspheres, followed by biotinylated detection antibody. Detection of the proteins was accomplished by incubation with phycoerythrin-labeled streptavidin. The resultant bead immuno-complexes were then read on a FLEXMAP3D (Luminex, TX, USA) with the instrument settings recommended by the manufacturer.

### Statistical Analysis

Luminex median fluorescence intensity (MFI) data were subjected to the quality control steps as described in our earlier study (15). Briefly, wells with individual bead counts <30, or bead CV >200 were flagged for exclusion. The coefficients of variation of replicate wells were also checked and wells with CV ≥25% were not included in further analyses. Concentration and MFI values for standards were log2 transformed prior to determination of the concentration using the standard curve (15). The log2-transformed data were tested for the normal distribution by plotting frequency histograms, prior to subsequent statistical analysis. The potential differences between T1D patients without any complication (T1D) and T1D patients with MA were initially examined using *t*-test. The pairwise correlation between individual protein levels was computed using Pearson correlation coefficient. The correlations between the protein levels were visualized using hierarchical clustering and presented as a heatmap. The effect of age and T1D duration on serum levels of each candidate molecule was determined using a linear regression including age or T1D duration as covariate on data stratified by sex and disease status. To examine the relationships between MA and the serum protein levels, logistic regression was used. Age, sex, T1D duration, hypertension (HTN), and dyslipidemia were included as covariates in a stepwise manner in the logistic regression.

To assess the odds ratios (ORs) for MA at different levels of each protein. Serum level for each protein was divided into five quintiles containing 20% MA patients in each quintile (20th percentile). The cutoff protein levels from MA patients were then used to count control subjects (T1D patients without MA) and MA subjects in each quintile. The first quintile was used as reference and OR for MA was calculated for the upper four quintiles. Pearson’s chi-squared test with Yates’ continuity correction was used to calculate the ORs. The chi-squared test for trend in proportions was used to calculate the *p*-value of overall trend.

Risk scores (equal to OR/quintile) were assigned to each subject based on individual protein levels. Hierarchical clustering and heatmap of risk scores were used to visualize the MA patients at high, medium, and low risk, based on these risk scores. To assess the OR, using a combination of proteins, the combined risk score of each subject was calculated by simply adding risk score from multiple proteins. The combined risk score was used to calculate ORs of having MA, for upper four quintiles using the first quintile as reference.

The receiving operator characteristic (ROC) curves were used to evaluate the ability of single proteins and multiprotein models to distinguish MA patients from controls. Sensitivity values of individual and combinations of proteins at different specificity thresholds (90; 95; 99; 100%) were computed. The utility of proteins as biomarkers was assessed using the area under curve (AUC) of the ROC curves for different models.

All *p*-values were two-tailed and a *P* < 0.05 value was considered statistically significant. All statistical analyses were performed using the R language and environment for statistical computing (R version 2.15.1; R Foundation for Statistical Computing; www.r-project.org).

## Results

### Ten Serum Proteins Significantly Elevated in MA Patients

**Figure 1:**
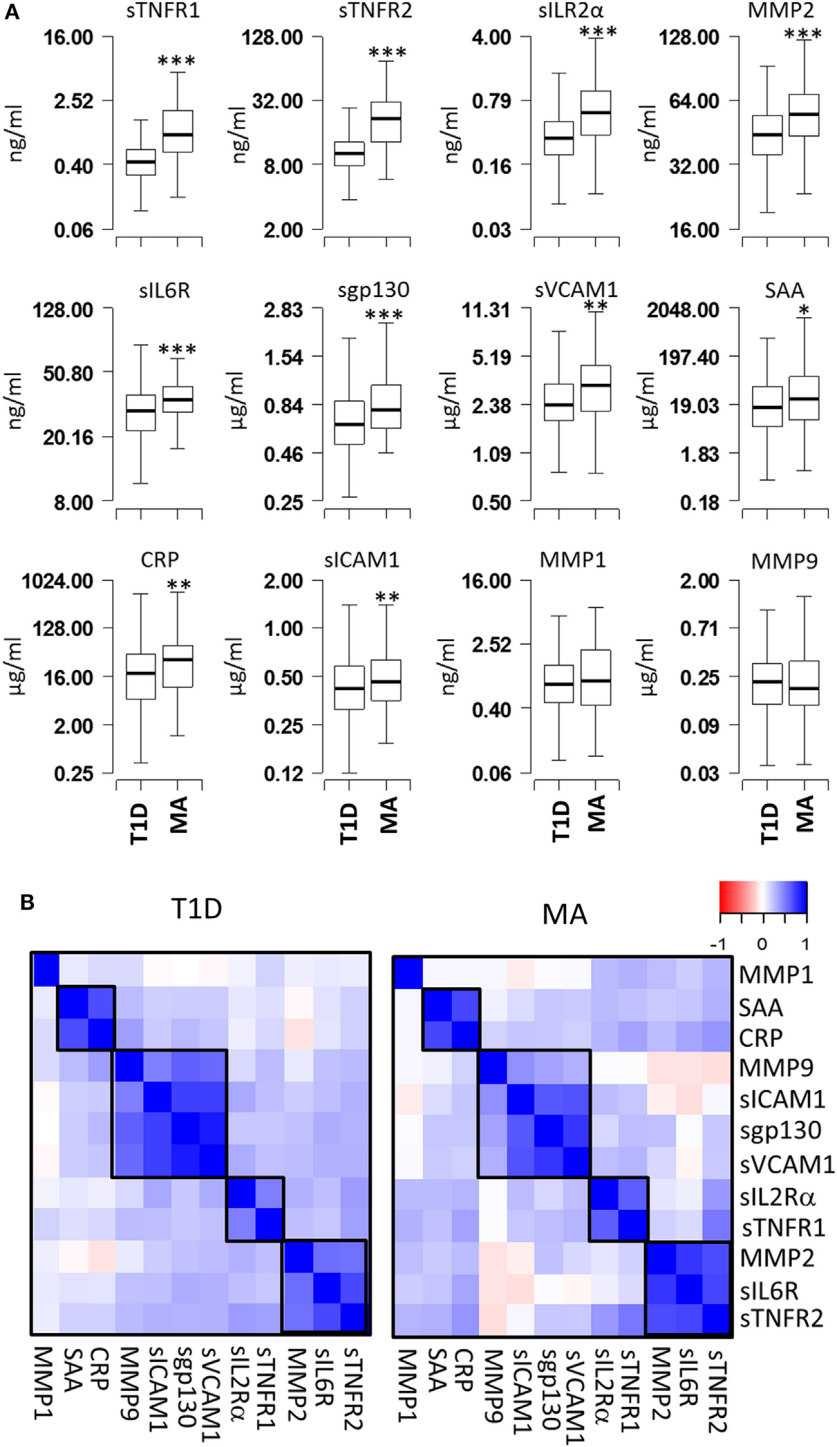
Comparison of serum protein levels and pairwise correlations. **(A)** Box plots showing the expression levels of 12 proteins in the microalbuminuria (MA) (*n* = 88) and nMA (*n* = 482) patient groups. **(B)** Heatmaps showing the pairwise correlation coefficients in type-1 diabetes (T1D) patients with and without MA. The correlation coefficients of proteins are clustered based on the hierarchical clustering. Boxes indicate groups of proteins with higher correlations. *** *P* < 0.000001, ** *P* < 0.01, and * *P* < 0.05.

Serum levels of 12 proteins (CRP, SAA, sICAM1, sVCAM1, MMP1, MMP2, MMP9, sIL2Rα, sIL6R, sgp130, sTNFR1, and sTNFR2) were measured in 483 T1D patients without MA (T1D) and 89 T1D patients with MA (MA) (Figure 1A). Comparison of the mean expression levels between T1D and MA groups revealed highly significant differences for six proteins (sTNFR1, sTNFR2, sIL2Rα, MMP2, sIL6R, and sgp130; *P* < 1 × 10^−5^) and moderately significant differences for four proteins (CRP, SAA, sICAM1, and sVCAM1; *P* < 0.05) (Table S1 in Supplementary Material). The remaining two proteins (MMP1 and MMP9) showed no statistically significant difference. We calculated correlations between each pair of the 12 proteins for T1D patients without complications and with MA separately. Hierarchical clustering of the correlation matrix defined four clusters of correlated proteins (Figure 1B). The first cluster includes sTNFR2, sIL6R, and MMP2 (*r* = 0.57–0.79); the second cluster includes sTNFR1 and sIL2Rα (*r* = 0.63); the third cluster consists of sgp130, sVCAM1, sICAM1, and MMP9 (*r* = 0.4–0.92); and the fourth cluster includes SAA and CRP (*r* = 0.66). These results indicate that a correlated group of proteins might have a common upstream regulator and correlated changes in these immunologically active proteins contribute synergistically to the pathogenesis of MA.

### Influence of Covariates on Serum Protein Levels

We next examined the potential influence of various covariates on serum protein levels in T1D patients with or without MA. The only significant correlations with age were found for MMP2 (T1D: *r* = 0.15, *P* = 0.0017; MA: *r* = 0.41, *P* < 1 × 10^−4^) and sTNFR2 (*r* = 0.09, *P* = 0.05 vs. *r* = 0.30, *P* = 0.004). The only significant correlations with the duration of diabetes were found for MMP2 (T1D: *r* = 0.23, *P* < 1 × 10^−5^; MA: *r* = 0.33, *P* < 0.01) and sTNFR2 (T1D: *r* = 0.17, *P* < 1 × 10^−3^) (Tables S2 and S3 in Supplementary Material). Since subject age and duration of diabetes are confounded covariates, it is difficult to distinguish the effects due to age vs. duration of diabetes. A small gender difference was observed for SAA (*F*/*M* ratio = 0.7, *P* < 0.01) and sTNFR1 (*F*/*M* ratio = 1.17, *P* < 0.01) only in T1D patients (Table S4 in Supplementary Material).

Even though there was no major impact of covariates on serum protein levels, we still adjusted for these covariates in logistic regression to rule out any confounding in the protein differences observed between MA and T1D patients (Table 1). Logistic regression analyses were carried out using protein concentration without any covariate adjustment (Model 1), then adjusting for age (Model 2), age and duration of diabetes (Model 3), and age, duration of diabetes, and gender (Model 4). Since HTN and dyslipidemia are also risk factors for MA, we adjusted for these two variables in a separate multivariate model which included age, duration of diabetes, and gender (Model 5). In these logistic regression analyses, 7 of the 12 proteins (sTNFR1, sTNFR2, sIL2Rα, MMP2, sgp130, sVCAM1, and sIL6R) showed significant associations with MA before and after adjusting for different covariates (Table 1).

**Table 1:** Odds ratios for microalbuminuria estimated using logistic regression.

Protein	Model 1	Model 2	Model 3	Model 4	Model 5
sTNFR1	5.47 (3.83–8.15)^¶^	4.80 (3.36–7.16)^¶^	4.58 (3.17–6.90)^¶^	4.78 (3.29–7.27)^¶^	4.03 (2.74–6.20)^¶^
sTNFR2	4.85 (3.29–7.36)^¶^	3.87 (2.62–5.91)^¶^	3.41 (2.30–5.20)^¶^	3.44 (2.32–5.26)^¶^	2.83 (1.87–4.42)^¶^
sIL2Rα	2.79 (2.14–3.70)^¶^	2.59 (1.98–3.45)^¶^	2.55 (1.92–3.44)^¶^	2.60 (1.96–3.51)^¶^	2.61 (1.92–3.61)^¶^
MMP2	2.30 (1.67–3.24)^¶^	1.85 (1.34–2.63)^‡^	1.51 (1.08–2.17)*	1.52 (1.09–2.19)*	1.56 (1.08–2.31)*
sgp130	1.87 (1.37–2.59)^‡^	1.83 (1.32–2.57)^‡^	1.77 (1.26–2.54)^†^	1.78 (1.26–2.56)^†^	1.90 (1.29–2.83)^†^
sVCAM1	1.69 (1.27–2.27)^‡^	1.61 (1.20–2.19)^†^	1.68 (1.23–2.33)^†^	1.68 (1.23–2.33)^†^	1.73 (1.23–2.45)^†^
CRP	1.52 (1.17–2.01)^†^	1.48 (1.12–1.98)^†^	1.57 (1.17–2.14)^†^	1.56 (1.16–2.13)^†^	1.33 (0.97–1.84)
sIL6R	1.72 (1.21–2.54)^†^	1.70 (1.17–2.55)^†^	1.68 (1.13–2.59)*	1.68 (1.13–2.58)*	1.58 (1.06–2.46)*
SAA	1.37 (1.07–1.77)*	1.24 (0.96–1.61)	1.39 (1.05–1.85)*	1.37 (1.04–1.83)*	1.17 (0.87–1.59)
sICAM1	1.34 (1.06–1.70)*	1.26 (0.98–1.61)	1.25 (0.96–1.62)	1.23 (0.95–1.60)	1.18 (0.90–1.56)
MMP1	1.22 (0.96–1.56)	1.11 (0.86–1.44)	1.22 (0.93–1.59)	1.22 (0.93–1.59)	1.10 (0.83–1.47)
MMP9	0.99 (0.79–1.27)	1.03 (0.81–1.35)	1.05 (0.8–1.39)	1.04 (0.80–1.39)	1.06 (0.8–1.46)

*Values presented are odds ratio (95% CI). Model 1 = protein concentration only, Model 2 = protein concentration + age, Model 3 = Model 2 + duration of T1D, Model 4 = Model 3 + gender, and Model 5 = Model 4 + hypertension and dyslipidemia*.

*P < 0.05, ^†^P < 0.01, ^‡^P < 0.001, and ^¶^P < 1 × 10^−5^.

### MA Associated with Increasing Serum Protein Levels

**Figure 2:**
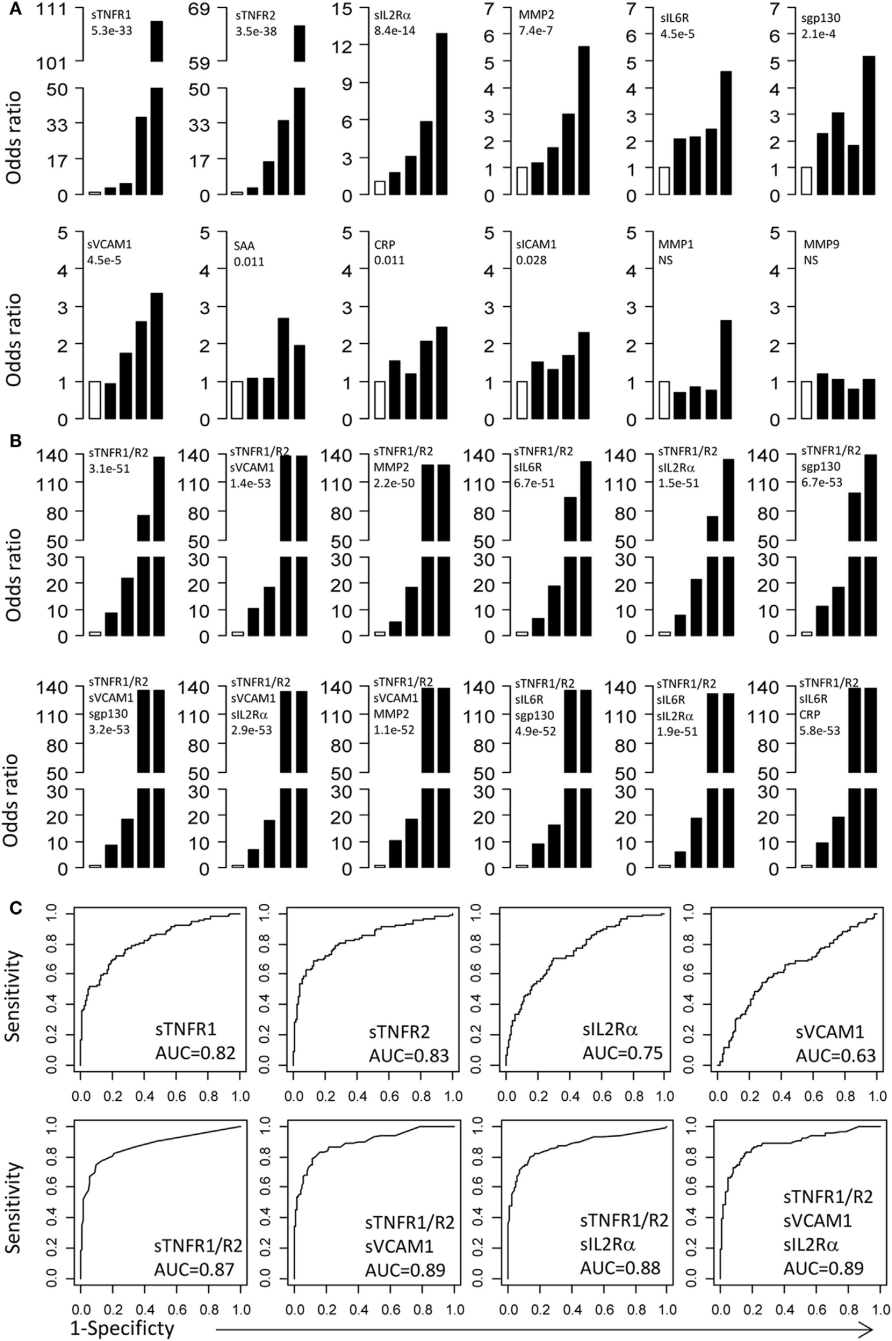
Odds ratios (OR) and receiver operating curves (ROC). **(A)** ORs associated with each of the top four quintiles compared with the bottom first quintile for each of the 12 individual proteins. The open bar represents the first quintile as reference (OR = 1). From left to right, each of the other four solid bars represents the second to fifth quintile (20% of the MA patients). Vertical axes are ORs. **(B)** ORs associated with the risk scores calculated based on different combinations of proteins. **(C)** ROC curves for selected proteins and protein combinations.

To examine the relationship between MA and serum proteins, we calculated the ORs for protein concentrations distributed into five quintiles. For each protein, the serum concentration in MA patients was divided into five quintiles of 20th percentile. Based on the quintile cutoff values determined from MA patients, T1D patients were also assigned to five groups. The first quintile was used as reference to calculate ORs for the second to fifth quintiles. The ORs with 95% CI and *p*-values for each protein are presented in Figure 2A and Table 2. The most important conclusion from these data is that MA is associated with increasing levels of 10/12 proteins measured in this study. The strongest association is observed with sTNFR1 (*P* < 1 × 10^−32^), which has an OR of 108.3 for the fifth quintile, and ORs of 36.1, 5.2, and 3.2 for the fourth, third, and second quintiles. Soluble TNFR2 has the second strongest association with ORs of 65.5 for the top quintile and ORs ranged from 3.1 to 34.7 for the second to fourth quintile (overall *P* < 1 × 10^−37^). Soluble IL2Rα is the third best protein with maximum OR of 12.9 (*P* < 1 × 10^−13^). MMP2, sgp130, sVCAM-1, and sIL6R have maximum ORs between 3 and 6 (Figure 2A; Table 2).

**Table 2:** Odds ratio (95% CI) of having microalbuminuria for each quintile.

Protein	Quintile 2OR (95% CI)	Quintile 3OR (95% CI)	Quintile 4OR (95% CI)	Quintile 5OR (95% CI)	Adj.*P*-trend
sTNFR1	3.24 (1.62–6.47)	5.16 (2.54–10.5)	36.11 (13.67–95.4)	108.34 (26.64–440.63)	5.3 × 10^−33^
sTNFR2	3.12 (1.56–6.24)	15.12 (6.80–33.6)	34.69 (13.49–89.24)	65.53 (21–204.44)	3.5 × 10^−38^
sIL2Rα	1.70 (0.86–3.37)	3.05 (1.51–6.16)	5.8 (2.77–12.15)	12.9 (5.64–29.49)	8.4 × 10^−14^
MMP2	1.19 (0.59–2.38)	1.75 (0.87–3.54)	3.02 (1.46–6.24)	5.55 (2.57–11.97)	7.4 × 10^−7^
sgp130	2.29 (1.10–4.75)	3.05 (1.45–6.41)	1.82 (0.88–3.74)	5.15 (2.37–11.19)	2.1 × 10^−4^
sIL6R	2.09 (1.03–4.22)	2.14 (1.06–4.33)	2.44 (1.2–4.96)	4.59 (2.18–9.64)	4.5 × 10^−5^
sVCAM1	0.94 (0.46–1.92)	1.73 (0.83–3.61)	2.59 (1.22–5.51)	3.34 (1.54–7.22)	4.5 × 10^−5^
SAA	1.07 (0.52–2.21)	1.07 (0.52–2.21)	2.68 (1.25–5.74)	1.95 (0.93–4.11)	0.011
CRP	1.54 (0.74–3.19)	1.19 (0.58–2.45)	2.07 (0.99–4.34)	2.45 (1.16––5.18)	0.011
sICAM1	1.51 (0.72–3.16)	1.31 (0.63–2.73)	1.68 (0.8–3.53)	2.29 (1.08–4.87)	0.028
MMP1	0.69 (0.34–1.39)	0.85 (0.42–1.72)	0.76 (0.38–1.53)	2.63 (1.22–5.67)	NS
MMP9	1.20 (0.57–2.53)	1.05 (0.50–2.20)	0.79 (0.38–1.63)	1.04 (0.50–2.17)	NS
sTNFR1 + sTNFR2	8.59 (4.16–17.75)	21.89 (9.61–49.85)	75.39 (24.29–234.01)	135.7 (33.39–551.45)	3.1 × 10^−51^
sTNFR1 + sTNFR2 + sVCAM1	10.27 (4.91–21.47)	18.6 (8.37–41.31)	137.63 (33.87–559.27)	137.63 (33.87–559.27)	1.4 × 10^−53^
sTNFR1 + sTNFR2 + MMP2	5.13 (2.55–10.34)	18.33 (8.19–41.03)	128.3 (31.57–521.48)	128.3 (31.57–521.48)	2.2 × 10^−50^
sTNFR1 + sTNFR2 + sIL6R	6.53 (3.21–13.30)	18.83 (8.41–42.15)	94.17 (27.37–324.04)	131.84 (32.44–535.82)	6.7 × 10^−51^
sTNFR1 + sTNFR2 + sIL2Rα	7.53 (3.67–15.44)	21.63 (9.5–49.26)	74.5 (24–231.26)	134.09 (33–544.93)	1.5 × 10^−51^
sTNFR1 + sTNFR2 + sgp130	11.31 (5.37–23.83)	18.64 (8.39–41.4)	98.54 (28.64–339.02)	137.96 (33.95–560.61)	6.7 × 10^−53^
sTNFR1 + sTNFR2 + sVCAM1 + sgp130	10.9 (5.19–22.9)	15.97 (7.32–34.83)	137.31 (33.79–557.97)	137.31 (33.79–557.97)	3.2 × 10^−53^
sTNFR1 + sTNFR2 + sVCAM1 + sIL2Rα	8.36 (4.05–17.24)	18.29 (8.23–40.63)	135.38 (33.31–550.15)	135.38 (33.31–550.15)	2.9 × 10^−53^
sTNFR1 + sTNFR2 + sVCAM1 + MMP2	7.01 (3.43–14.32)	17.99 (8.1–39.97)	133.13 (32.76–541.04)	133.13 (32.76–541.04)	1.1 × 10^−52^
sTNFR1 + sTNFR2 + sIL6R + sgp130	9.03 (4.35–18.72)	16.51 (7.53–36.21)	135.38 (33.31–550.15)	135.38 (33.31–550.15)	4.9 × 10^−52^
sTNFR1 + sTNFR2 + sIL6R + sIL2Rα	6.13 (3.02–12.44)	18.74 (8.37–41.94)	131.2 (32.28–533.22)	131.2 (32.28–533.22)	1.9 × 10^−51^
sTNFR1 + sTNFR2 + sIL6R + CRP	9.36 (4.51–19.44)	19.52 (8.72–43.68)	136.67 (33.63–555.38)	136.67 (33.63–555.38)	5.8 × 10^−53^

*OR, odds ratio; NS, not significant*.

*Quintile 1 was used as a reference. Individual protein concentrations were assigned the OR for the quintile and then summed to combine the risk scores for individual proteins*.

### Protein Combinations Defining Three Subsets of MA Patients

Since multiple serum proteins are associated with MA, we attempted to examine the combined effect of these proteins on MA. For this purpose, we calculated risk scores for each subject by adding the quintile ORs from multiple proteins and then examined association between MA and the risk scores. We first examined the risk scores based on the two best proteins, sTNFR1 and sTNFR2. The combination of sTNFR1 and sTNFR2 improved the highest OR values (OR = 135.7), suggesting an increase in proportion of MA subjects in the top quintile (*P* < 1 × 10^−50^) (Figure 2B and Table 2). We then examined 10 models of three-protein combinations by adding, each time, one of the remaining 10 proteins to sTNFR1 and sTNFR2. The three-protein models did not improve the maximum OR of the sTNFR1/2 model (OR = 135.7); however, the ORs associated with the fourth quintiles improved for all models and reached the levels of the fifth quintile for two models (sTNFR1/2 + sVCAM1 and sTNFR1/2 + MMP2). Subsequently, we examined two sets of four-protein models. The first set of four-protein models includes nine combinations (TNFR1/2 + sVCAM1 + one of the nine remaining) and the second set of four-protein models includes eight combinations (TNFR1/2 + sIL6R + one of the eight remaining). In general, these 17 models performed similarly as the better three-protein models (Figure 2B; Table 2).

Examination of the best three- and four-protein models suggests the existence of three subsets of MA patients. The MA patients in the top two quintiles (fourth and fifth) had extremely high risk scores (range 74.5–137.9, mean = 126.3) and the MA patients in the second and third quintiles have moderate risk score (range 5.1–21.9, mean = 13.5), while the MA patients in the bottom quintile have low risk score (reference group with OR = 1) (Figure 2B; Table 2).

### Potential Utility of TNF-α/IL6 Proteins as MA Biomarkers

Receiving operator characteristic curves were used to evaluate the potential utility of these serum proteins as MA biomarkers (Figure 2C). The AUC for individual proteins is reasonable for two proteins (sTNFR1 = 0.82 and sTNFR2 = 0.83), but the AUC values for the other proteins are poor (<0.75). ROC curves for protein combinations were also evaluated using the combined risk scores. Combinations of three or four proteins that contain both sTNFR1 and sTNFR2 improved the AUC values to 0.87–0.89 (Figure 2C). These protein combinations were able to achieve 100% specificity with 14.8–18% sensitivity, 99% specificity with 37.3–39.8% sensitivity, or 95% specificity with 60.8–62.5% sensitivity (Table S5 in Supplementary Material).

## Discussion

This study demonstrated significant increases in 10 of the 12 examined serum proteins in MA patients compared with T1D patients without MA. Although the concentrations for two proteins (MMP2 and sTNFR2) are slightly but significantly correlated with subject age and duration of diabetes, the differences between MA and T1D patients cannot be accounted for by any of the examined covariates. The strongest associations with MA in this study were observed with the two soluble receptors of TNF-α (sTNFR1 and sTNFR2) and moderate associations were observed with several other soluble receptors (sIL2Rα, sIL6R, and sgp130) and soluble adhesion molecules (sVCAM1 and sICAM1). Using the risk score system developed in this study, combinations of these proteins allowed the definition of three subsets of MA patients with distinct patterns of the TNF-α/IL6 profiles. The identification of subsets of patients may allow the design of novel intervention strategies for different MA patients. Compared with the previous studies (16), we here show that the top 40% and the middle 40% of the MA patients have increased inflammation through the TNF-α/IL6 pathway. Blocking this pathway in these patients may ameliorate their clinical outcomes. In contrast, intervention of the TNF-α/IL6 pathway may not be effective for the bottom 20% of the patients who do not have elevated inflammation through the TNF-α/IL6 pathway. The pathogenesis in these patients may be related to the other molecules that are yet to be identified. Our risk score system may also prove to be very useful to develop biomarkers for the identification of MA patients or even identification of high-risk T1D patients for the development of MA. Indeed, serum levels of sTNFR1 and sTNFR2 in the fourth quartile have recently been found to be predictive of an early renal function loss in T1D and T2D patients (13, 16). It will be interesting to determine whether the prediction can be improved using our risk score system.

TNF-α is a pro-inflammatory cytokine, implicated in microvascular and structural changes in the kidneys of diabetic patients acting *via* TNFR1 and TNFR2 receptors. The observed elevation of the soluble TNF receptors supports the hypothesis that high concentrations of sTNFRs are pro-inflammatory by acting as the slow release reservoirs of TNF-α, which may be responsible for the chronic inflammatory state as observed in nephropathy (17). TNF-α signaling *via* TNF receptors leads to the activation of Th1-lymphocytes and endothelium by producing receptors and adhesion molecules required for abnormal migration and retention of leukocytes and lymphocytes in kidneys (18). TNFR1 is expressed ubiquitously on the surface of all cells and is directly related to the increased apoptosis *via* caspase-8 pathway (19, 20). On the other hand, TNFR2 is expressed on T-lymphocytes upon activation and is required for the cell survival and activation (21). The elevated levels of sTNFRs may reflect kidney damage *via* several mechanisms involving cell death, production of reactive oxygen species by activated leukocytes, and structural changes to renal tissues.

Elevated levels of sIL2Rα have been found in virus mediated and IgA nephropathy and Balkan nephropathy (14, 22). This is the first time that elevated levels of sIL2Rα have been reported in T1D patients with MA. Elevated levels of sIL2Rα predict the renal outcome in IgA nephropathy patients. The elevation of sIL2Rα and sTNFR2, both found on activated T cells, suggests the presence of an activated T-cell phenotype in MA patients, observed previously in patients with MA (23, 24). Influx of activated T cells in kidneys is associated with changes in glomerular structure and albumin excretion in T1D patients, as well as T1D patients with proteinuria, although the results are debatable (23, 24). The exact clinical relevance of activated T cells in kidneys of T1D patients is not very clear; it appears that damage to the kidneys occurs by activating the infiltrated macrophages. Activated macrophages in turn release reactive oxygen species, cytokines such as IL1, TNF-α, complement factors, and metalloproteinases, all of which promote renal injury (18).

The second component of the inflammatory cytokine network involves the IL6 pathway. The effect of IL6 on target cells is mediated by a complex receptor system, composed of IL6R and a signal-transducing glycoprotein (gp130). The increased concentration of soluble IL6R may be suggestive of metabolic syndrome and insulin resistance in T1D patients (25). The increase in IL6 signaling observed in T1D patients (26, 27) modulates immune response through the expansion of pathogenic Th17 cells and inhibition of generation of Foxp3 + T-regulatory cells is associated with T1D autoimmunity (28–30). It has been shown that Th17 cells contribute to inflammation during chronic kidney progression (31). The expression of IL6R is limited to the activated macrophages, lymphocytes, and leukocytes, whereas IL6 signaling in endothelial cells and other cell types occurs *via* trans-signaling through interaction of IL6/sIL6R complex with the surface bound gp130 molecule. The elevated levels of sgp130 may suggest higher gp130 on the surface of renal endothelium and renal cells along with the activated leukocytes and lymphocytes in kidneys of MA subjects leading to increased IL6 trans-signaling and kidney injury (32, 33). Indeed, end-stage renal disease patients undergoing hemodialysis have higher expression of membrane gp130 in PBMCs, and increased spontaneous release of membrane bound gp130 (32).

Apart from the direct effect on kidneys and activation of lymphocytes, signaling *via* TNFRs and IL6R also elicits inflammatory responses by the liver and vascular endothelial cells. Under increased inflammatory stimulus, hepatic cells respond by eliciting acute phase reaction through production of CRP and SAA. Elevated levels of the acute phase proteins (CRP and SAA) are known to be deposited in the glomerular endothelium as well as in the cytoplasm of tubules. It is hypothesized that these deposits promote inflammation *via* the release of IL6, IL1, and TNF-α and chemotactic molecules by the renal cells (18, 19, 34, 35). TNF-α and CRP both upregulate the expression of ICAM1 and VCAM1 on the surface of glomerular endothelial cells (35, 36). The elevated levels of adhesion molecules (sICAM1 and sVCAM1) suggest the presence of subclinical endothelial dysfunction, which may cause increased recruitment and migration of leukocytes in the kidneys of MA patients as observed in an earlier study (19). Increased signaling *via* TNFRs and IL6R is also involved in the pathophysiology of kidneys including nephropathy *via* production of MMPs in renal tissues (37). MMP2, a zinc metalloprotease, is involved in the shedding of receptors and activation of cytokines and chemokines (38). Overexpression of MMP2 in kidneys of mice has been shown to cause structural abnormalities similar to those observed in human MA patients (39). The elevated serum levels of inflammatory, matrix metalloproteinase, and endothelial markers observed in this study reflect an increase in TNF-α and IL6-mediated secondary inflammation and endothelial dysfunction which may result in abnormal changes in renal physiology, integrity, and retention and migration of leukocytes in the kidneys of T1D patients.

In conclusion, we found significant increase in 10 serum proteins of TNF-α/IL6 pathway, which has been previously implicated in the initiation and progression of MA. Using multiple proteins and a novel risk score system, we identified three subsets of MA patients with distinct inflammatory profile. The identification of subsets may allow the design of novel intervention strategies for each subset of patients.

## Author Contributions

SP and J-XS were involved with conception of the project. SP was responsible for data acquisition and analysis. AS and SP were responsible for data analysis. WZ, DH, LS, BB, SA, JR, and RS contributed to clinical samples. All authors contributed to writing and editing of the manuscript.

## Conflict of Interest Statement

The authors declare that the research was conducted in the absence of any commercial or financial relationships that could be construed as a potential conflict of interest.
