# Supplementary material for: Proteins of TNF-α and IL6 Pathways Are Elevated in Serum of Type-1 Diabetes Patients with Microalbuminuria
Source: Front Immunol. 2018 Jan 31;9:154. doi: 10.3389/fimmu.2018.00154 (PMC5797770; doi:10.3389/fimmu.2018.00154)
Supplement: Supplementary file 1 [file Table_1.DOCX]

**Supplementary Table S1**: Demographics and serum measurements data for study subjects.

| **Clinical**  **Variable** | **T1D**  **(n=483)** | **MA**  **(n=89)** | **p-val** | **Fold change** |
| --- | --- | --- | --- | --- |
| Sex (%F) | 51 | 57 | NS |  |
| Age (Years) | 39.15 (20.0-73.8) | 48.58 (22.0-73.8) | 1.7x10^-9^ |  |
| Duration of T1D (Years) | 18.19 (0.0-57.6) | 29.14 (0.6-53.1) | 5.7x10^-13^ |  |
| Dyslipidemia (%) | 22.2 | 48.3 | 5.0x10^-7^ |  |
| Hypertension (%) | 12 | 57.3 | 6.8x10^-23^ |  |
| Hemoglobin (mg/dL) | 14.3+1.5 | 14.1+1.7 | 5.4x10^-5^ |  |
| Albumin (g/dL) | 4.4+0.4 | 4.1+0.5 | 0.001 |  |
| LDL (mg/dL) | 93.7+27.5 | 81.8+37.5 | NS |  |
| Total Cholesterol (mg/dL) | 174.2+34.3 | 175.3+35.4 | NS |  |
| Triglycerides (mg/dL) | 90.1+67.4 | 101.8+35 | 0.0008 |  |
| HDL (mg/dL) | 61.9+17.8 | 55.6+13.8 | NS |  |
| Serum Creatinine (mg/dL) | 0.9+0.2 | 1.5+1 | 2x10^-7^ |  |
| HbA1c (%) | 7.8+1.1 | 8+1 | NS |  |
| Systolic BP (mmHg) | 117.9+9.5 | 127.2+11.3 | 2x10^-7^ |  |
| Diastolic BP (mmHg) | 73.6+6.4 | 74.5+6.1 | NS |  |
| BUN (mg/dL) | 13.4+4.5 | 22+16.6 | 8x10^-9^ |  |
| MicroAlbumin/Creatnine (mg/g)* | 5.6 (5.1-8.8) | 93.7 (31.9-297.7) | 1.7x10^-15^ |  |
| sTNFR1 (ng/ml) | 0.49+1.8 | 1.55+2.2 | 1.5x10^-17^ | 3.17 |
| sTNFR2 (ng/ml) | 10.1+1.7 | 21.7­+2.3 | 6x10^-13^ | 1.70 |
| sIL2Ra (ng/ml) | 0.3+1.9 | 0.6+2.1 | 2.4x10^-12^ | 2.02 |
| sVCAM1 (μg/ml) | 2.4+1.8 | 3.1+1.7 | 3.9x10-04 | 1.62 |
| sIL6R (ng/ml) | 28.3+1.5 | 34.4+1.4 | 5.05E-06 | 1.20 |
| sgp130 (μg/ml) | 0.7+1.6 | 0.8+1.5 | 6.52E-05 | 1.23 |
| MMP2 (ng/ml) | 43.3+1.4 | 55.3+1.4 | 1.30E-05 | 1.28 |
| SAA (μg/ml) | 16.9+4.5 | 26.1+174.7 | 0.017 | 1.55 |
| CRP (μg/ml) | 14.7+4 | 24+4.1 | 0.004 | 1.63 |
| MMP1 (ng/ml) | 0.8+2.5 | 0.9+2.8 | NS | 1.13 |
| sICAM1 (μg/ml) | 0.4+1.7 | 0.5+1.8 | 0.0062 | 1.25 |
| MMP9 (μg/ml) | 0.2+1.9 | 0.2+184.6 | NS | 0.94 |

Proportion of females/males, presence of dyslipidemia and hypertension is presented as percentages. Age and duration of T1D data is presented as mean (range). Clinical values presented are means + SD. UACR: urinary albumin to-creatinine ratio (median [IQR]). *Mann-Whitney test. T1D: T1D patients without microalbuminuria, MA: T1D patients with microalbuminuria

**Supplementary Table S2:** Correlations between serum levels and age.

|  | T1D | | MA | |
| --- | --- | --- | --- | --- |
| **Protein** | **r** | **p-val*** | **r** | **p-val*** |
| CRP | 0.030 | 0.549 | 0.101 | 0.366 |
| SAA | 0.128 | 0.010 | 0.224 | 0.043 |
| MMP1 | 0.120 | 0.011 | 0.194 | 0.070 |
| MMP2 | 0.149 | 1.7E-03 | 0.414 | 6.8E-05 |
| MMP9 | -0.080 | 0.111 | 0.009 | 0.933 |
| sICAM1 | 0.042 | 0.399 | 0.085 | 0.456 |
| sVCAM1 | -0.020 | 0.696 | 0.248 | 0.025 |
| sgp130 | -0.043 | 0.395 | 0.202 | 0.069 |
| sIL6R | -0.040 | 0.404 | 0.148 | 0.171 |
| sIL2Rα | 0.041 | 0.389 | 0.128 | 0.236 |
| sTNFR1 | 0.089 | 0.060 | 0.234 | 0.028 |
| sTNFR2 | 0.094 | 0.048 | 0.303 | 0.004 |

*p-values presented are not adjusted.

T1D: T1D patients without microalbuminuria, MA: T1D patients with microalbuminuria

**Supplementary Table S3:** Correlation between serum levels of protein and duration of diabetes

|  | T1D | | MA | |
| --- | --- | --- | --- | --- |
| **Protein** | **r** | **p-val*** | **r** | **p-val*** |
| CRP | -0.029 | 0.556 | -0.031 | 0.783 |
| SAA | -0.063 | 0.209 | 0.148 | 0.184 |
| MMP1 | -0.013 | 0.780 | -0.123 | 0.254 |
| MMP2 | 0.228 | 1.30E-06 | 0.334 | 0.002 |
| MMP9 | -0.027 | 0.592 | -0.128 | 0.250 |
| sICAM1 | 0.054 | 0.278 | -0.094 | 0.407 |
| sVCAM1 | -0.041 | 0.416 | 0.035 | 0.752 |
| sgp130 | 0.007 | 0.896 | 0.139 | 0.212 |
| sIL6R | -0.042 | 0.377 | 0.074 | 0.498 |
| sIL2Rα | 0.084 | 0.077 | 0.075 | 0.487 |
| sTNFR1 | 0.106 | 0.025 | 0.055 | 0.613 |
| sTNFR2 | 0.170 | 3.16E-04 | 0.139 | 0.200 |

*p-values presented are not adjusted. T1D: T1D patients without microalbuminuria, MA: T1D patients with microalbuminuria

**Supplementary Table S4:** Gender differences in serum protein levels

|  | T1D |  |  |  | MA |  |
| --- | --- | --- | --- | --- | --- | --- |
| Protein | F / M | p value |  |  | F / M | p value |
| CRP | 0.84 | 0.059 |  |  | 0.84 | 0.222 |
| SAA | 0.70 | **0.002** |  |  | 0.70 | 0.557 |
| MMP1 | 0.97 | 0.410 |  |  | 0.97 | 0.336 |
| MMP2 | 1.06 | 0.063 |  |  | 1.06 | 0.451 |
| MMP9 | 0.96 | 0.201 |  |  | 0.96 | 0.222 |
| sICAM1 | 0.95 | 0.677 |  |  | 0.95 | 0.213 |
| sVCAM1 | 1.02 | 0.408 |  |  | 1.02 | 0.650 |
| sgp130 | 1.05 | 0.150 |  |  | 1.05 | 0.825 |
| sIL6R | 1.06 | 0.087 |  |  | 1.06 | 0.493 |
| sIL2Rα | 1.08 | 0.389 |  |  | 1.08 | 0.029 |
| sTNFR1 | 1.17 | **0.008** |  |  | 1.17 | 0.020 |
| sTNFR2 | 0.98 | 0.641 |  |  | 0.98 | 0.419 |

T1D: T1D patients without microalbuminuria, MA: T1D patients with microalbuminuria

**Supplementary Table S5:** Area under the curve and sensitivity of ROC curves before and after grouping the protein levels into quintiles

|  | AUC |  | Specificity (%) | | | |
| --- | --- | --- | --- | --- | --- | --- |
| Protein | (95% CI) | p val | 90 | 95 | 99 | 100 |
| sTNFRI | 0.82(0.79 - 0.85) | 7.90E-22 | 52.81 | 50.56 | 29.21 | 13.48 |
| sTNFRII | 0.83(0.80 - 0.86) | 4.20E-23 | 62.92 | 53.93 | 19.10 | 0 |
| sIL2Ra | 0.75(0.72 - 0.78) | 6.50E-14 | 38.20 | 29.21 | 6.74 | 4.49 |
| sVCAM1 | 0.63(0.60 - 0.66) | 1.70E-04 | 21.68 | 12.05 | 0 | 0 |
| sTNFRI+sTNFRII | 0.87(0.85 - 0.89) | 2.30E-28 | 75.21 | 60.77 | 37.30 | 14.77 |
| sTNFRI+sTNFRII+sVCAM1 | 0.89(0.87 - 0.91) | 2.50E-31 | 73.97 | 61.36 | 39.77 | 14.77 |
| sTNFRI+sTNFRII+sIL6R | 0.88(0.86 - 0.90) | 7.80E-30 | 71.00 | 61.06 | 38.63 | 18.18 |
| sTNFRI+sTNFRII+sVCAM1+sIL6R | 0.89(0.87 - 0.91) | 2.50E-31 | 77.27 | 62.50 | 39.77 | 18.18 |

Individual protein concentration was converted into OR/quintile based Risk scores and then summed to combine the data for combinations
